# Analysis of the potential for immunosuppression of infants with in-utero and breast milk exposure to antitumor necrosis factor alpha agents: are all agents equally safe?

**DOI:** 10.1097/JW9.0000000000000220

**Published:** 2025-09-08

**Authors:** Marita Yaghi, Ajay S. Dulai, Nina R. Haddad, Emi M. Murase, Jenny E. Murase

**Affiliations:** a Department of Dermatology, Larkin Community Hospital, South Miami, Florida; b Integrative Skin Science and Research, Sacramento, California; c Mount Sinai Medical Center, Miami Beach, Florida; d Department of Biological Sciences, University of California, Davis, Davis, California; e Department of Dermatology, Palo Alto Foundation Medical Group, Mountain View, California; f Department of Dermatology, University of California, San Francisco, California

**Keywords:** breastfeeding, immunosuppression, pregnancy, psoriasis, tumor necrosis factor inhibitors, vaccination

## Abstract

**Objective:**

To assess the safety of tumor necrosis factor inhibitors (TNFi) during pregnancy, specifically in relation to infant infection rates, vaccine efficacy, and vaccine-associated adverse events in infants exposed to TNFi in utero and through breast milk.

**Data Sources:**

A comprehensive literature review was conducted using PubMed and Google Scholar. The review included retrospective studies, prospective studies, and systematic reviews published until June 2024, focusing on TNFi exposure during pregnancy and breastfeeding.

**Study Selections:**

Studies reporting on infant infection rates, vaccination outcomes, and adverse events following in-utero or breastfeeding exposure to TNFi agents, including certolizumab pegol, adalimumab, infliximab, etanercept, and golimumab. Narrative reviews were excluded, while systematic reviews were considered.

**Results:**

The review indicates that gestational exposure to TNFi does not increase the risk of severe infections in infants. Additionally, no significant rise in infections was found in infants exposed to TNFi through breast milk. Vaccination outcomes, including efficacy and adverse events, were comparable between exposed and unexposed infants. However, caution is advised when administering the Bacille Calmette–Guerin vaccine to infants exposed to TNFi, particularly within the first year of life.

**Conclusion:**

TNFi appear to be safe during pregnancy and breastfeeding. The data indicate that TNFi does not increase the risk of severe infections or vaccine complications in infants, with the exception of the Bacille Calmette–Guerin vaccine, for which a more cautious approach is recommended. These findings reinforce the use of TNFi in women with inflammatory conditions who are pregnant or breastfeeding, providing benefits to both maternal and fetal health.

**What is known about this subject in regard to women and their families?**

- Untreated or poorly controlled inflammatory conditions such as psoriasis and psoriatic arthritis during pregnancy pose significant risks to both maternal and fetal health.
- For the mother, these conditions can increase the risk of complications like gestational hypertension, pre-eclampsia, gestational diabetes, and postpartum depression.
- For the baby, risks include fetal complications such as low birth weight, preterm delivery, congenital abnormalities, and intrauterine growth restriction.
- The use of tumor necrosis factor inhibitors (TNFi) during pregnancy has been controversial, as there have been concerns about potential risks to the developing fetus, including immune suppression, which may lead to increased infections and altered immune responses to vaccinations.

**What is new from this article as messages for women and their families?**

- Our work provides an in-depth summary of the available evidence regarding the safety of TNFi use during pregnancy and breastfeeding, showing that these biologics, when used during pregnancy, do not significantly increase the risk of infections or compromise the infant’s ability to respond to vaccines.
- We highlight that exposure to TNFi, either in utero or through breast milk, does not result in a higher incidence of serious infections in infants, providing reassurance for women who require TNFi therapy during pregnancy.
- We recommend delaying the Bacille Calmette–Guerin vaccine for infants exposed to TNFi in utero and suggest monitoring anti-TNF levels in exposed infants to ensure their safety. This information is crucial for healthcare providers and women with inflammatory conditions who are considering or are already undergoing TNFi treatment during pregnancy, offering evidence-based guidance to manage the risks while maintaining the benefits of treatment.

## Introduction

Untreated or poorly managed inflammatory conditions such as psoriasis (PsO) and psoriatic arthritis (PsA) can pose significant risks to both the mother and baby.^1^ These risks include fetal complications such as congenital abnormalities, premature delivery, intrauterine growth restriction, low birth weight, and macrosomia.^2–5^ For the mother, severe uncontrolled PsO/PsA increases the risk of pregnancy-related complications, including gestational hypertension, preeclampsia, gestational diabetes, and postpartum depression.^6^ Beyond these physical health risks, untreated disease can profoundly impact the mother’s quality of life, as PsO is associated with increased prevalence of depression and anxiety.^7,8^

Tumor necrosis factor (TNF) is a pro-inflammatory cytokine that has become the target of various biologic therapies.^9^ TNF initiates a cascade of pro-inflammatory cytokines, prostaglandins, and other lipid signal transduction mediators that regulate macrophage and T-cell proliferation, differentiation, and apoptosis.^9^ Due to this pivotal role that TNF plays in the inflammatory cascade, TNF inhibitor (TNFi) therapy has become an effective treatment in various autoimmune and inflammatory conditions. Currently available agents include certolizumab pegol (CZP), infliximab (INF), adalimumab (ADA), golimumab (GOM), and etanercept (ETC). TNFi have become instrumental in the treatment of inflammatory conditions, namely PsO, PsA, and hidradenitis suppurativa.^10^ TNFi have revolutionized the treatment of PsO/PsA as they can lead to complete skin lesion clearance, with improved clinical response in cases of skin and joint involvement compared with methotrexate.^11,12^ Furthermore, TNFi usage reduces the incidence of PsO-associated cardiovascular comorbidities such as myocardial infarctions.^13^ On the other hand, by inhibiting TNF, a key player in the immune response to pathogens, these biologics disrupt the normal immune cascade.^14^ A meta-analysis found that anti-TNF treatment resulted in an increased risk of serious infections, including tuberculosis, bacterial infections, and opportunistic fungal infections.^15^ Furthermore, TNFi agents may harbor an elevated risk of lifetime malignancies, but no definite association has been established.^16,17^

The role of TNF during pregnancy remains poorly understood. While some research suggests that it contributes to early fetal and placental development, other research implicates TNF as a marker capable of impairing blastocyst growth.^18,19^ Most importantly, TNFi can cross from maternal to fetal circulation via binding of the Fc region of antibodies to the FcRn transporter on the placenta, which is more active during the third trimester.^20,21^ CZP is the only exception to this rule, given that it lacks an Fc region and has a unique polyethylene glycol portion of the antibody, initially designed to protect it from destruction by the immune system. As such, CZP is not actively transported across the placenta during pregnancy and is the only biologic agent with clinical trial data in its label supporting its use in both pregnancy and breastfeeding.^22^ On the other hand, INF, ADA, ETC, and GOM are found at higher levels in cord blood. This difference is of utmost importance when we consider the potential adverse effects of fetal transfer of TNFi. The primary concern of TNFi usage consists of the risk of immunosuppression, which may manifest as increased prevalence of infections and impaired vaccine response. For instance, there has been a report of infantile death following administration of the Bacille Calmette–Guerin (BCG) vaccine in an infant exposed to TNFi in utero for maternal Crohn’s disease.^23^ While the baby was born at 36 weeks with no complications, BCG vaccine administration at 3 months of age led to infant demise at 4.5 months of age from disseminated BCG.

The assessment of TNFi safety during pregnancy is important to gauge the safety of these therapeutics when caring for pregnant women who suffer from inflammatory skin conditions. Herein, we summarize available evidence regarding infection rates and vaccination safety outcomes in infants exposed to TNFi.

## Methods

Published literature was surveyed for data on human studies reporting infection rates and vaccination safety outcomes in infants exposed to CZP, ADA, INF, GOM, and ETC in-utero or through breastfeeding for the treatment of mothers with various autoinflammatory diseases, including but not limited to PsO, PsA, rheumatoid arthritis, ankylosing spondylitis, and Crohn’s disease. Articles published until June 2024 were identified using relevant search terms on PubMed and Google Scholar. The literature included encompassed clinical trials and primary data presentations that reported specific infection rates and vaccination safety outcomes. Narrative literature reviews were excluded, and systematic reviews were included. The Oxford 2011 Levels of Evidence Table was used to assess the quality of each included reference.^24^

Unpublished data from pregnancy exposure databases were also requested from pharmaceutical companies commercializing TNFi.

## Results

**Fig. 1.**
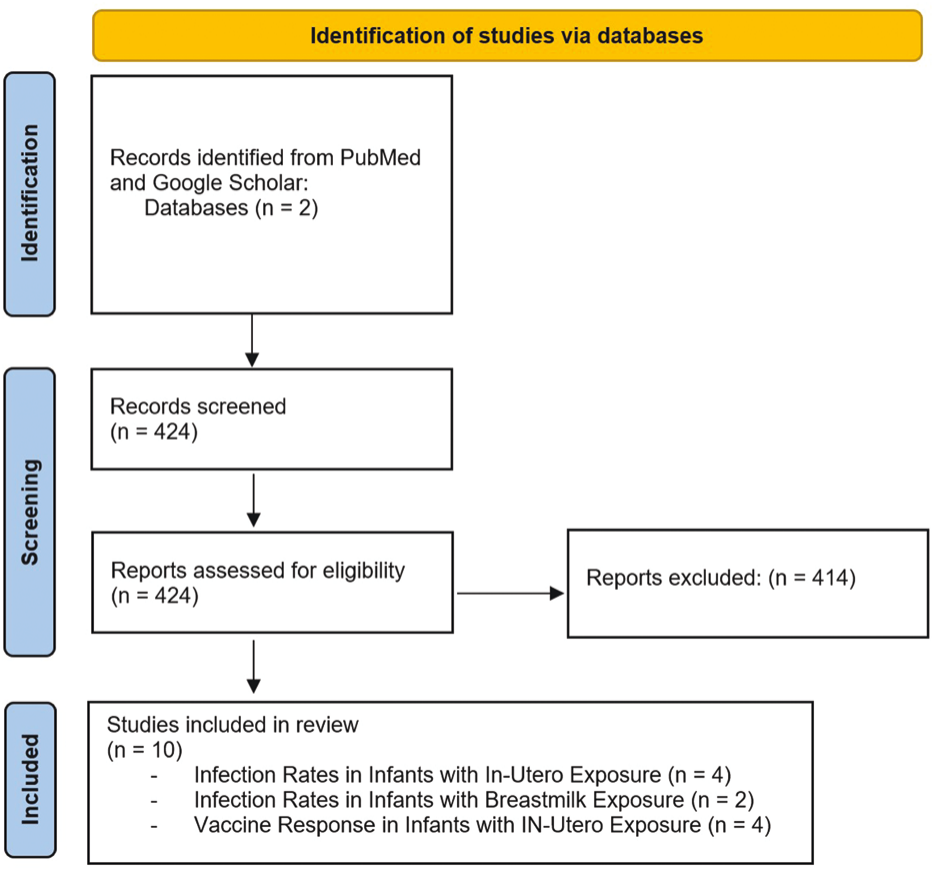
PRISMA diagram of article screening and selection process.

A total of 424 articles were selected and assessed for eligibility. Ten articles met the inclusion criteria and were included in this review (Fig. 1). One article had a levels of evidence score of 4, four articles had a score of 3, and 5 articles had a score of 2.

### Infantile infection rates and TNFi exposure

#### Infant Exposure to TNFi In-utero

Four major studies were found reporting on infection rates in infants secondary to exposure to TNFi in utero (Table 1).

**Table 1:** Literature summary on infection rates in infants following in-utero exposure to TNFi

Reference	Study type	Number of patients	TNFi investigated	Reported outcomes	Results	Oxford 2011 levels of evidence
Mahadevan et al. (2018)^25^	Prospective multicenter cohort	Group 1: TNFi: N = 642 Group 2: TNFi + Thiopurines: N = 227	INF; ADA; GOM; CZP	Infections	Group 1: OR = 0.92 (95% CI [0.70–1.20]) Group 2: OR = 0.93 (95% CI [0.66–1.32])	2
Dernoncourt et al. (2022)^26^	Retrospective pharmacovigilance database	N = 464	ADA; INF; GOM; CZP; ETC	Infections	• All conditions ROR = 1.32 (95% CI [1.00–1.74]) • IBD ROR = 2.75 (95%CI [1.20–6.28])	2
Chaparro et al. (2018)^27^	Retrospective multicenter cohort	N = 388 • TNFi alone: 75% • TNFi + thiopurines: 25%	ADA; INF; CZP (0.3%)	Outcome 1: Infections Outcome 2: Severe infections	Exposed vs. nonexposed Outcome 1: 4.1% vs 0.9%, *P* = .002 Outcome 2: Univariate analysis: 1.6% vs 2.8% (*P* = .2) Multivariate analysis: HR: 1.2 (0.8–1.8)	3
Luu et al. (2018)^28^	Retrospective national cohort	N = 1,457	INF; ADA; GOM; CZP	Infections	aOR = 0.89 (95% CI [0.76–1.05])	2

ADA, adalimumab; CI, confidence interval; CZP, certolizumab pegol; ETC, etanercept; GOM, golimumab; HR, hazard ratio; INF, infliximab; OR, odds ratio; ROR, reporting odds ratio; TNFi, tumor necrosis factor inhibitors.

Between 2007 and 2019, pregnant women with inflammatory bowel disease (IBD) were enrolled in a prospective, observational, multicenter study across the United States, constituting the pregnancy inflammatory bowel disease and neonatal outcomes registry.^25^ Among 1,490 completed pregnancies, 43% were exposed to TNFi monotherapy and 15% were exposed to both anti-TNFs and thiopurines. Controlling for preterm birth, maternal age, and disease activity, use of biologic or combination therapy was not associated with increased risk of any infection in the first year of life.

Using the World Health Organization’s (WHO) large retrospective pharmacovigilance database analysis (VigiBase), more than 9,500 individual case safety reports, both maternal and fetal, from 1968 to 2021, were analyzed.^26^ The study excluded all paternal exposure, exposure through breastfeeding, exposure secondary to COVID-19, and cases with concomitant exposure to teratogenic drugs. Focusing on TNFi exposure in-utero, 464 infants were included. Reporting odds ratio revealed similar risk of infection overall, comparing infants exposed to TNFi and those unexposed. Stratification for IBD revealed a significantly higher reporting odds ratio of 2.75, suggesting that maternal disease influences infection outcomes.

In the TEDDY study, a European multicenter retrospective study, 841 charts were reviewed of infants from mothers with IBD treated with TNFi agents or thiopurines.^27^ Around 46% (N = 399) of this sample was exposed to TNFi, and 25% of those were exposed to thiopurines. Rates of infantile infection in the gestational TNFi-exposed group were higher compared to nonexposed (4.1% vs 0.9%; *P* = .002). However, a similar incidence of severe infections was noted on both univariate and multivariate analysis in nonexposed vs exposed children: 1.6% vs 2.8% per person-year (*P* = .2); hazard ratio: 1.2 (0.8–1.8).

An exposed retrospective cohort was conducted on the French national health system database “Système National d’Information Inter-Régimes de l’Assurance Maladie” (SNIIRAM), surveying 11,275 pregnancies between 2011 and 2014.^28^ Data on the 1,457 pregnancies with exposures to TNFi revealed no increased odds of developing infections.

Overall, the majority of published data suggests that there is no increased incidence of infection during the infancy of individuals with in-utero TNFi exposure; however, one source does suggest that there was an increased risk of nonsevere infections.

#### Infants exposure to TNFi through breastmilk

Focusing on exposure through breastmilk, 2 studies reporting on infection rates in infants were found (Table 2): one large study assessed the risk with exposure to anti-TNF drugs^29^ and another study focused on CZP alone.^30^

**Table 2:** Literature summary on infection rates in infants following exposure to TNFi through breastmilk

Reference	Study type	Number of patients	Medications investigated	Reported outcomes	Results	Oxford 2011 levels of evidence
Matro et al. (2018)^29^	Prospective multicenter cohort	1. TNFi: N = 243 2. TNFi + Thiopurines: N = 67 3. Control: N = 202	INF ADA GOM CZP	Infections at 12 m	1. 37% 2. 39% 3. 45% Overall: *P* > .99	3
Clowse et al. (2017)^30^	Prospective postmarketing cohort	N = 17	CZP	Infections	47% - consistent with reported epidemiologic rates	2

ADA, adalimumab; CZP, certolizumab pegol; ETC, etanercept; GOM, golimumab; INF, infliximab; TNFi, tumor necrosis factor inhibitors.

A multicenter prospective study of women with IBD and their infants found that infants exposed to biologics were not more likely to have an infection in the first 12 months of life compared with unexposed infants.^29^

Similar results were reported in uncontrolled postmarketing CZP studies based on the CRADLE postmarketing phase 4 trial.^30^ Seventeen children with breastfeeding exposure were followed during the first 6 months of life. However, 47% developed infections, which was found to be not significantly different when compared with unexposed infants. There were no reported severe infections requiring hospitalization.

Currently published literature is consistent in finding no association of changes to infantile infection rates based on exposure through breastmilk.

### Infantile vaccination outcomes and TNFi exposure

#### Infant exposure to TNFi in utero

Looking into the potential effect of TNFi on vaccination outcomes, we found a total of 4 studies, including 2 prospective studies, 1 of which focused on CZP monotherapy, 1 retrospective analysis, and 1 systematic review (Table 3). All these studies reported on in-utero exposure to TNFi.

**Table 3:** Literature summary on vaccine response in infants following in-utero exposure to TNFi

Reference	Study type	Number of patients	Medications investigated	Reported outcomes	Results	Oxford 2011 levels of evidence
Meroni et al. (2018)^31^	Prospective observational	N = 12	CZP	Serologic response to: 1. DTaP, IPV, HBV 2. Hib	1. 99% 2. 93.2% vs. 100% (general population)	4
Beaulieu, et al. (2018)^32^	Prospective multicenter	Total surveyed: N = 179 • TNFi: N = 153 • No TNFi: N = 26 Total blood samples: N = 33	INF; ADA; GOM; CZP	Outcome 1: Titers for HiB & Tdap Outcome 2: Rotavirus safety outcomes	Outcome 1: No differences in humoral responses Outcome 2: No serious adverse reactions	3
Zerbo et al. (2022)^33^	Retrospective cohort	N = 660	INF; ADA; GOM; CZP	Safety of live attenuated vaccines Outcome 1: MMR Outcome 2: Rotavirus	For both: Similar to unexposed cohort Outcome 1: Rare AESI Outcome 2: Mild AE	2
Goulden et al. (2022)^34^	Systematic review	N = 276 Group 1: BCG: N = 215 Group 2: Rotavirus: N = 46 Group 3: MMR: N = 12	INF; ADA; GOM; CZP	Safety of live attenuated vaccines	Group 1: No reactions Group 2: 7 mild reactions Group 3: 8 reactions, 1 leading to death	3

ADA, adalimumab; CZP, certolizumab pegol; GOM, golimumab; INF, infliximab; TNFi, tumor necrosis factor inhibitors.

A prospective observational study over a 4-year time frame was performed in 12 newborns exposed to CZP during pregnancy.^31^ The serologic immune responses to the diphtheria, tetanus, acellular pertussis vaccine, the inactivated poliovirus vaccine, hepatitis B virus vaccine, and inactivated Haemophilus influenzae type b vaccine were assessed by ELISA and neutralization assays, comparing the results to the general population. Adequacy and noninferiority of immune response for these children were observed compared with the response in the general healthy population.

From the pregnancy inflammatory bowel disease and neonatal outcomes registry, a multicenter, prospective observational study in the United States, 179 pregnant women completed the vaccination survey, and umbilical cord blood samples were collected from 33 children.^32^ Looking at serologic response to Haemophilus influenzae type b and tetanus toxoid vaccines, no differences in immune response were observed in the children exposed to anti-TNFs in utero as compared with the general population. Additionally, looking at survey data, Rotavirus vaccine was administered without serious adverse reactions, and with mild events occurring at a rate of that in the general population.

A retrospective cohort study among children born from 2006 to 2017 at 6 sites within the Vaccine Safety Datalink, a collaboration between the Centers for Disease Control and Prevention and 9 integrated health care systems across the United States, looked at the data of 582,759 infants, of which 660 were exposed to TNFi.^33^ Examining the adverse events following exposure to the measles, mumps, and rubella (MMR) vaccine, reported outcomes showed adverse events that were similarly rare between biologically exposed and unexposed children. Focusing on outcomes in those who received the rotavirus vaccine, a few diagnoses of diarrhea, bloody stools, and vomiting were observed. Rates were similar between children exposed and unexposed to anti-TNFs. In all children, there were no cases of febrile seizure, cerebellar ataxia, hepatitis, pneumonitis, acute disseminated encephalomyelitis, encephalitis and myelitis, or intussusception.

A systematic review of live vaccine outcomes in infants exposed to TNFi looked at data reporting on MMR, rotavirus, and BCG. A total of 276 infants were included.^34^ Overall, no reactions following MMR were observed, and 7 mild reactions to rotavirus vaccination were reported. However, although only 8 reactions to BCG were noted, 1 report included death.

Generally, data on vaccination outcomes in infants exposed to TNFi suggest that there is no difference in vaccination efficacy or adverse events when compared with unexposed infants. However, the cases of death following BCG vaccination warrant further investigation and caution when administering the BCG vaccine to exposed infants.

## Discussion

Our results indicate that exposure to TNFi, whether being in utero or through breast milk, appears to be safe. Overall, the studies that assessed risks of infection did not find a higher risk in infants exposed to TNFi.^26–30,35^ While 1 study did find an increased rate of infantile infections, stratification by severity showed no significant increase in serious infections.^33–36^ As for vaccine outcomes, adequate responses to regularly administered vaccines were noted, and similar expected adverse reactions were noted when comparing those of infants who were exposed to TNFi and those who were not. The only noted adverse unexpected reaction was the death of an infant exposed to the BCG vaccine, for which we recommend delaying the vaccination or measuring the anti-TNF blood level before vaccine administration.^23^

Our findings align with previous data, which showed that newborns with a history of exposure to TNFi should follow a standard vaccination schedule for inactive vaccines.^37^ Furthermore, the current vaccination guidelines of the European Crohn’s and Colitis Society recommend the avoidance of live vaccines within the first 6 to 12 months of life or until blood levels of the biologic are no longer detectable.^38^ Our findings support this recommendation. Our results also show that in infants exposed to TNFi, the rotavirus vaccine was administered without serious adverse reactions and with mild events consistent with the general population. These findings align with the American College of Rheumatology recommendations of the administration of rotavirus vaccine within the first 6 months of life for people exposed to TNFi, as opposed to rituximab (a CD-20 inhibitor), in which the administration of rotavirus is recommended to be done after the first 6 months of life.^39^

The use of prospective and retrospective cohort studies in this study offers substantial benefits in understanding the safety of drug exposure during pregnancy. This longitudinal approach minimizes recall bias and enhances the reliability of our findings. By using large datasets that control for confounding factors, the included studies effectively examine the rare exposure of TNFi use during pregnancy, providing a robust framework for investigating its effects on infant health.

The absence of randomized controlled trials in this research could impact the robustness of the conclusions due to ethical challenges in conducting trials with pregnant women. Future studies could monitor adverse outcomes in women who require the drug during pregnancy and conduct pharmacokinetic studies to better understand exposure levels in infants, enhancing clinical guidelines.

The findings from this study have significant implications for clinical practice and the development of future guidelines concerning the use of TNFi during and postpregnancy. Given that our findings suggest that these drugs are safe for infants who were exposed to TNFi in utero or during breastfeeding, we support the usage of TNFi in women of childbearing potential, pregnant women, and women who are breastfeeding, as this can reduce systemic inflammation, maternal complications, and adverse fetal outcomes.^2–6^

## Conclusion

In brief, infants exposed to TNFi in utero or through breast milk appear to have similar rates of infections as compared with their unexposed peers, as well as adequate and appropriate responses to routine vaccinations. CZP monotherapy showed no superiority to other agents in this regard.

Based on the current literature, we believe a standard vaccination schedule for vaccines in infants of mothers who were administered TNFi during pregnancy can be followed. The BCG vaccine is the only exception, and we believe delaying its administration after the first year of life or measuring the biologic blood levels before vaccinating the infant are safer practices. As new research is published in this field, we should continue to evaluate the potential effects of gestational TNFi therapy.

Given the current evidence available, dermatologists should not be deterred from prescribing TNFi for women with inflammatory skin conditions during their reproductive journey, as these treatments can significantly improve quality of life and outcomes for both mother and child. However, dermatologists should still ensure detailed consultation with the patient, which describes the potential risks and benefits of TNFi treatment during pregnancy. By demonstrating a positive safety profile through various cohort studies, the research supports the potential for broader use of this drug in pregnant populations when the severity of the maternal disease warrants systemic therapy.

## Conflicts of interest

The authors made the following disclosures: JEM is on the Speakers Board for Galderma, UCB, and Sanofi-Regeneron, and has served on Advisory Boards for UCB, Galderma, Arcutis, Sanofi-Regeneron, and Bristol-Myers Squibb, and provides dermatologic consulting services for UCB, AbbVie, Sanofi-Regeneron, and UpToDate. MY receives research support from the HS Foundation. The other authors have no relevant conflicts of interest to declare.

## Funding

None.

## Study approval

N/A.

## Author contributions

MY and JEM: Conceptualization, research, statistics, editing, proofreading. EMM: Statistics, editing and first draft. AD: First draft and subsequent drafts. NH: Subsequent drafts. All authors read and approved final version.
